# A Scalable Topical Vectored Vaccine Candidate against SARS-CoV-2

**DOI:** 10.3390/vaccines8030472

**Published:** 2020-08-24

**Authors:** Mohammed A. Rohaim, Muhammad Munir

**Affiliations:** Division of Biomedical and Life Sciences, Faculty of Health and Medicine, Lancaster University, Lancaster LA1 4YG, UK; m.a.rohaim@lancaster.ac.uk

**Keywords:** newcastle disease virus, recombinant vaccine, SARS-CoV-2, CoVID-19, pandemic

## Abstract

The severe acute respiratory syndrome-coronavirus 2 (SARS-CoV-2) caused an ongoing unprecedented global public health crises of coronavirus disease in 2019 (CoVID-19). The precipitously increased death rates, its impact on livelihood and trembling economies warrant the urgent development of a SARS-CoV-2 vaccine which would be safe, efficacious and scalable. Owing to unavailability of the vaccine, we propose a de novo synthesized avian orthoavulavirus 1 (AOaV-1)-based topical respiratory vaccine candidate against CoVID-19. Avirulent strain of AOaV-1 was engineered to express full length spike (S) glycoprotein which is highly neutralizing and a major protective antigen of the SARS-CoV-2. Broad-scale in vitro characterization of a recombinant vaccine candidate demonstrated efficient co-expression of the hemagglutinin-neuraminidase (HN) of AOaV-1 and S protein of SARS-CoV-2, and comparable replication kinetics were observed in a cell culture model. The recombinant vaccine candidate virus actively replicated and spread within cells independently of exogenous trypsin. Interestingly, incorporation of S protein of SARS-CoV-2 into the recombinant AOaV-1 particles attributed the sensitivity to anti-SARS-CoV-2 antiserum and more prominently to anti-AOaV-1 antiserum. Finally, our results demonstrated that the recombinant vaccine vector stably expressed S protein after multiple propagations in chicken embryonated eggs, and this expression did not significantly impact the in vitro growth characteristics of the recombinant. Taken together, the presented respiratory vaccine candidate is highly attenuated in primates per se, safe and lacking pre-existing immunity in human, and carries the potential for accelerated vaccine development against CoVID-19 for clinical studies.

## 1. Introduction

An outbreak of pneumonia erupted in a Chinese seafood market in Wuhan during late 2019 and within a month of its origin, on 30 January 2020, a Public Health Emergency of International Concern was declared by the World Health Organization (WHO) due to its high human-to-human transmission. Within the next month, the outbreak of coronavirus disease in 2019 (CoVID-19) soared among communities unprecedentedly and spread across the globe and a pandemic was declared on 11 March 2020 by the WHO. A large proportion (~80%) of CoVID-19 infected patients showed only moderate symptoms which led to a staggering rate increase in the global spread of the infection. The acute respiratory distress syndrome, manifested in ~20% of CoVID-19 patients, causing substantial case fatality rates especially in elderly and frail people with co-morbidities [1,2].

The severe acute respiratory syndrome-coronavirus 2 (SARS-CoV-2), the causative agent of the ongoing CoVID-19 pandemic, belongs to the family *Coronavirdae* within the *Betacoronavirus* (β-CoV) genus. Similar to SARS-CoV and Middle Eastern respiratory syndrome-related coronavirus (MERS-CoV), SARS-CoV-2 carries a single stranded linear RNA genome with positive polarity [3]. The genome consists of four structural proteins including envelope (E), spike (S), membrane (M), and nucleocapsid (N), 16 non-structural proteins (nsp 1–16) and multiple accessary proteins. Amongst these viral proteins, the S protein constitutes a major protective antigen that elicits highly specific antibodies mediated immune responses [3]. Therefore, the S protein remained the primary vaccine markers against coronaviruses.

Currently, there is no registered drug or vaccine available to curb the pandemic; however, multiple vaccines using a range of technologies are being developed, or pre-clinically or clinically being investigated [4]. Amongst these, an inactivated vaccine has elicited strong antibodies which can neutralize multiple SARS-CoV-2 strains and can partially or fully protect macaques against SARS-CoV-2 challenge [5]. A chimpanzee adeno (ChAd)-vectored vaccine, expressing the full-length S gene of SARS-CoV-2, elicited humoral and cell-mediated responses in rhesus macaques [6]. However, it failed to fully alleviate clinical signs in vaccinated macaques albeit reduced severity and protection against pneumonia. The ChAdOx1 nCoV-19 also failed to reduce the viral replication in the nose, highlighting the potential spread of SARS-CoV-2 through sneezing even in vaccinated people [6]. Recently, clinical data demonstrated the induction of antibodies and T-cell responses in humans by both ChAdOx1 and adjuvanted killed vaccines [7,8,9]. Several novel mRNA and DNA vaccines are being trialed with promising early results in pre-clinical and clinical studies [10,11]. However, it remains to be determined if these immune responses protect vaccinated individuals against natural SARS-CoV-2 infection.

Owing to multifaceted advantages, the avian orthoavulavirus 1 (AOaV-1) proposes a potential vaccine vector against SARS-CoV-2. Specifically, AOaV-1 (represented by a type species, Newcastle disease virus, NDV) are exclusively cytoplasmic viruses and therefore the viral gene segments are not integrated into the host genome which raises their safety profile. Since these vectors lack natural recombination, the expression of transgenes is genetically stable. Additionally, AOaV-1 can infect multiple species of animals; the vaccines can be produced in chicken embryonated eggs and multiple cell lines using protocols and infrastructure currently being applied for influenza virus vaccines [12]. Given these and other features, apathogenic strains of AOaV-1 have been used as live attenuated vaccines against multiple viruses including influenza, SARS, human immunodeficiency virus [13,14], human parainfluenza, rabies [15], Nipah disease [16], Rift Valley fever [17], Ebola and highly pathogenic H5N1 [18,19]. Importantly, AOaV-1 has appeared to be safe and effective in mice [19], dogs [15], pigs [16], cattle [20,21], sheep [20], African green and rhesus monkeys [22,23] and humans [24,25,26,27]. Notably, the natural hosts of AOaV-1 are birds and the vector is antigenically distinct from common human pathogens. Therefore, no pre-existing immunity exists in humans which makes it an ideal vector to deliver transgene effective and safely.

In the present study, we de novo designed an AOaV-1 vector and generated a recombinant vaccine candidate by expressing full-length codon-optimized S protein of the SARS-CoV-2 at a pre-optimized gene junction. This topical respiratory vaccine candidate was fully characterized in vitro. Based on the infection and spreadability within cells, its sensitivity to anti-AOaV-1 and anti-SARS-CoV-2 neutralizing antibodies, its replication kinetics and stability in chicken embryonated eggs, the rAOaV-1-SARS-CoV-2 is a scalable topical vectored vaccine candidate against SARS-CoV-2 to be tested for safety and immunogenicity in animal studies.

## 2. Materials and Methods 

### 2.1. Cells and Viruses

The AOaV-1-wt vaccine vector was rescued as described previously [28], passaged twice on African green monkey kidney (VERO) cells, and in embryonated chicken eggs. Vero cell line was obtained from the American Type Culture Collection (ATCC, Manassas, VA, USA). These cells were grown in Dulbecco’s minimal essential medium (DMEM) containing 10% fetal bovine serum (FBS). Fertile embryonated chicken eggs were purchased from Henry Stewart & Co Ltd., Fakenham, UK.

### 2.2. Generation of Recombinant AOaV-1 Expressing S Protein of SARS-CoV-2

The genome of AOaV-1 encodes six structural genes in the order of nucleocapsid (N), phosphoprotein (P), matrix (M), fusion (F), hemagglutinin-neuraminidase (HN) and RNA dependent RNA polymerase (RdRP, also known as L). The transcription of the linear gene starts from gene start (GS) and ends at the gene-end (GE). Between the GS and GE, there is an intergenic sequence (IG). The foreign gene can be inserted at any of the gene junction (IG) with variable levels of transcription; however, recent studies have assessed optimal gene expression when inserted between P and M gene [14,28,29,30]. This arrangement of the gene is identical among all AOaV-1s.

In order to offer a competitive reverse genetic system as a novel vaccine vector, an avirulent strain of AOaV-1 was used carrying a lentogenic-like cleavage site and pathogenicity (patent pending on the technology). The full length antigenomic sequence of AOaV-1, originally isolated from asymptomatic wild birds, was partially de novo synthesized (NBS Biologicals, Huntingdon, UK) using a novel sequence modification approach. Rest of the genome length mainly constituting around the P gene was cloned using overlapping PCRs. The entire cassette was then shuttled into the TVT7R (0,0) (Addgene Plasmid #98631). In order to avoid incorporation of extra non-viral gene residues into the transcribed minigenome and efficient transcription of the gene, an autocatalytic and “rule of six” adhered HHRz was introduced at the 5′-end and hepatitis delta virus ribozyme (HdvRz) at the 3′ end of the antigenome.

An expression cassette for the full-length S gene was first in silico generated, containing the Kozak sequence, GE, GS, IG and the ORF for the full length S gene was codon-optimized for *Homo sapiens* codon-usage and inserted into the unique *PmeI* site between the P and M genes, which was originally preserved during the cloning of complete antigenome. The construct was named rAOaV-1-SARS-CoV-2 whereas the AOaV-1 without the insertion of the foreign gene (AOaV-1-wt) was used as infection control throughout the study. The orientation of the inserted S gene was confirmed by the nucleotide sequence analysis at the time of cloning as well as during the propagation of the vector in cells and chicken embryonated eggs.

The rAOaV-1-SARS-CoV-2 and AOaV-1-wt were used to rescue the infectious viruses as described previously [28] with substantial modifications. Briefly, Vero cells were infected with modified vaccinia Ankara (MVA) expressing the T7 polymerase at a multiplicity of infection 1.0 for 6 h. These cells were transfected with Lipofectamine 2000 using rAOaV-1-SARS-CoV-2 and AOaV-1-wt backbones as well as supporting N, P and L gene-expression plasmids (ratio of 1:0.8:0.4:0.1) for 72 h. After 3 days post-infection, cells and cell supernatants were mixed and freeze-thawed three times at −80 °C before inoculation into 8-day-old embryonated chicken eggs. After an additional three days, individual eggs were screened using hemagglutination assay and real-time PCR as we described before [31,32,33]. Successfully rescued isolates were further propagated to generate viral stock and for in vitro characterization.

### 2.3. Propagation of Viruses in Eggs and Cells 

The infectivity of the recombinant virus and parental wild type strains were characterized using a standard hemagglutination assay (HA). The 50% tissue infectious dose (TCID_50_) assay on Vero cells in 96-well plates, was performed and calculated following standard procedures [31,32,34].

### 2.4. Western Blotting

To confirm expression of the viral protein, Vero cells were infected with recombinant and wild type viruses at a multiplicity of infection (MOI) of 0.1 as we described earlier [35]. Cell lysates were collected at 24 h post infection and subjected to Western blot. Briefly, after 24 h post-infection, cells were washed once with phosphate buffered saline (PBS) followed by adding 100 μL ice-cold NP40 lysis buffer (completed with protease inhibitors cocktail) per well and incubated for 30 min on ice. All cell lysates were denatured at 98 °C for 8 min, proteins were separated by sodium dodecyl sulphate polyacrylamide gel electrophoresis (SDS-PAGE), and subsequently transferred to nitrocellulose membranes [35]. The cell lysates were subjected for centrifugation at 13,000 rpm for 30 min at 4 °C and the supernatants were incubated in sample loading buffer 2× (Biorad) containing 10% β-mercaptoethanol for 5 min at 98 °C, and separated on a 10% sodium dodecyl sulfate–polyacrylamide gel electrophoresis (SDS-PAGE), and proteins were transferred onto polyvinylidene difluoride (PVDF) membranes. The S and HN viral proteins were detected by incubation with primary antibodies (1:500 dilution). After probing with primary antibodies, the blots were incubated with peroxidase-conjugated species-specific secondary antibodies (Abcam, Cambridge, MA, USA) and visualized by chemiluminescence (Chemidoc, BioRad, Hercules, CA, USA), as we performed earlier [35].

### 2.5. Immunofluorescence

The expression of the S and HN proteins in recombinant virus-infected cells were evaluated by immunofluorescence assays using confocal microscopy. Vero cells grown on coverslips in 24-well plates were infected with wild type or recombinant viruses or viruses pre-incubated with antisera for 24 h. After fixing the cells with 4% paraformaldehyde in PBS for 30 min, washed with PBS, and permeabilized with 0.1% Triton X-100 for 10 min. After blocking of the cells with 5% bovine serum albumin (BSA) in PBS, they were incubated with monoclonal antibody (mAb) [36] to probe HN or S or both proteins. Binding of primary antibodies were visualized using Alexa 488 α-rabbit and 568 α-mouse secondary antibodies (Invitrogen, Loughborough, UK). The S and HN proteins expression were analyzed through fluorescence for wild-type and recombinant viruses compared to mock infected cells. The 4′,6-diamidino-2-phenylindole (DAPI) was used to stain cell nuclei and the images were captured using a Zeiss confocal laser-scanning microscope (Zeiss, Kohen, Germany). Digital images were processed using Adobe illustrator software, and the same parameters were applied to the whole image area.

### 2.6. Sensitivity of rAOaV-1-SARS-CoV-2 and AOaV-1-wt to Antisera

A total of 5 × 10^2^ TCID_50_ of AOaV-1-wt and rAOaV-1-SARS-CoV-2 was mixed with either anti-AOaV-1 sera (heat-inactivated and diluted to 1:100) or mouse SARS-CoV-2 anti-sera (dilution 1:10) or with plain mouse anti-sera as mock neutralization. These mixtures were incubated for 1 h at 37 °C before infection of Vero cells grown on coverslips in 24-wells plates. After 1 h of adsorption, the supernatant was removed, and cells were washed three times with PBS and supplemented with DMEM without serum. After 24 h post-infection, cells were fixed with paraformaldehyde and immunofluorescence staining was performed using anti-HN antibodies against AOaV-1 as described above. Images were captured using a Zeiss confocal laser-scanning microscope (Zeiss, Kohen, Germany) and processed using Adobe illustrator software using identical parameters on all frames.

### 2.7. Stability and Growth Characteristics of NDV Constructs Using Real-Time PCR

The stability of recombinant vaccine candidate compared to parental wild type was grown sequentially in embryonated chicken eggs for at least 5 passages. RNA was extracted from the allantoic fluid of recombinant and wild type after the first and fifth passages in eggs using the QIAamp Viral RNA Mini Kit (Qiagen, Manchester, UK). The real-time qRT-PCR was performed using SuperScript III Platinum One*-*Step qRT*-*PCR Kit to detect the NDV M gene [33], which enabled the calculation of viral genome copies that was plotted against hours post-infection for each of the viruses to produce standard curves. To investigate the growth properties of the recombinant viruses, Vero cells were infected in 6-well plates at a MOI of 0.1 after being washed twice with PBS and an adsorption time of 60 min. Cell supernatants were harvested 0, 6, 12, 24 and 48 h post infection (p.i). Viral titers (50% tissue culture infectious dose, TCID_50_) were calculated by immunofluorescence assay (IFA) using primary antibodies against viral proteins and Alexa Fluor 488 α-rabbit and Alexa Fluor 586 α-mouse as secondary antibodies (Invitrogen), respectively.

### 2.8. Statistical Analysis

Virus-infected and control groups were compared using Student’s *t*-test. All statistical analyses and figures were conducted in the GraphPad Prism (GraphPad Software, La Jolla, CA, USA).

## 3. Results

### 3.1. Design and Construction of AOaV-1-SARS-CoV-2 Vaccine Candidate

**Figure 1:**
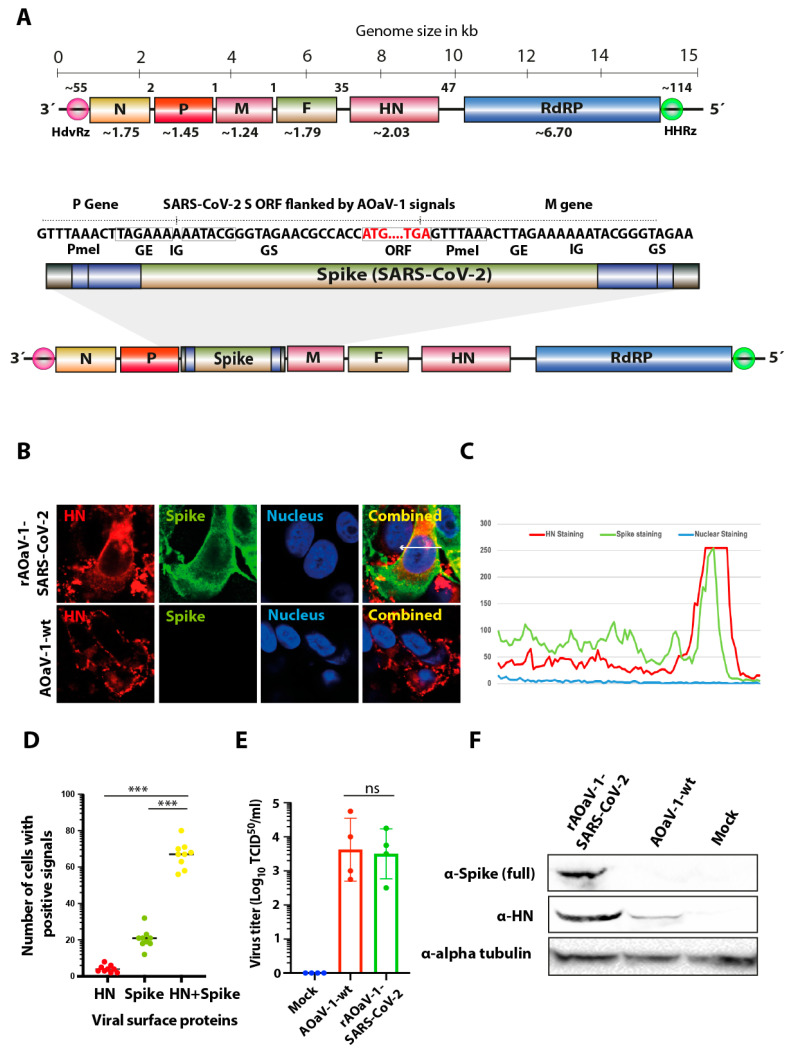
Construction, rescue and characterization of rAOaV-1-SARS-CoV-2 vaccine candidate. (**A**) The full-length ORF for S gene of SARS-CoV-2 was over-hanged with required transcriptional signals (GE, GS, IG) and inserted in between P and M genes. The rough gene size is mentioned below each gene, the division of the genome across the length and number of nucleotides in intergenic region is displayed at the top of the schema of the rAOaV-1 genome. (**B**) Vero cells were infected with the rAOaV-1-wt or rAOaV-1-SARS-CoV-2 and stained for the expression of the HN (red) or S (green) proteins. The co-expression of both surface proteins is colored yellow in combined images and was marked with the arrow. (**C**) Quantitative co-expression profile is marked with arrow and shown in the line chart. (**D**) A total of nine microscopic fields were scanned for the presence of HN or S or both proteins. A significantly higher proportion of HN+S expressing cells were identified. (**E**) A comparable replication of rAOaV-1-wt or rAOaV-1-SARS-CoV-2 in Vero cells indicating the competitive replication of rAOaV-1 even after the expression of the foreign S gene. (**F**) Western blot analysis for the expression of the HN protein, indicating the active replication of the rAOaV-1 and S protein indicating the rAOaV-1-SARS-CoV-2, indicating the replication competence of the recombinant virus. Alpha tubulin was used as loading control. *** indicates statistically significant difference with *p* < 0.005, and ns indicates non-significant.

An avirulent strain of AOaV-1 was used to construct a vaccine candidate against SARS-CoV-2. The full length antigenomic sequence of AOaV-1, originally isolated from asymptomatic wild birds, was de novo synthesized. To facilitate minigenome transcription, an autocatalytic and “rule-of-six” adhered hammerhead ribozyme sequence was introduced in both 5′ and 3′-ends of the antigenome (Figure 1A).

An expression cassette containing the Kozak sequence, GE, GS, IG and the ORF for the full-length S gene was codon-optimized for *Homo sapiens* codon usage and inserted into the unique *PmeI* site between the P and M genes, which was originally preserved during the cloning of complete antigenome (Figure 1A). The construct was named rAOaV-1-SARS-CoV-2 whereas the AOaV-1 without the insertion of the foreign gene (AoaV-1-wt) was used as infection control throughout the study. The orientation of the inserted S gene was confirmed by the nucleotide sequence analysis.

### 3.2. Rescue of Recombinant Vaccine and Evaluation of the Spike Gene Expression of SARS-CoV-2

The rAOaV-1-SARS-CoV-2 and AOaV-1-wt were rescued in Vero cells and propagated in 8-day-old embryonated chicken eggs. Screening of multiple individually inoculated eggs, using real-time PCR and hemagglutination assays, has successfully identified rescued rAOaV-1-SARS-CoV-2 viruses (Figure S1) which were used to fully characterize in the presented study.

Expression of the S protein was confirmed by indirect confocal immunofluorescent staining of rAOaV-1-SARS-CoV-2-infected Vero cells. Counter-staining of the HN protein of AOaV-1 and S protein of SARS-CoV-2 confirmed co-expression of the surface protein in rAOaV-1-SARS-CoV-2-infected Vero cells, whereas only HN protein expression was observed in AOaV-1-wt-infected cells (Figure 1B). Graphical profile of the expression intensities confirmed co-expression of both surface proteins (Figure 1C) where a vast majority of rAOaV-1-SARS-CoV-2-infected cells expressed both HN and S proteins simultaneously (Figure 1D). 

Both recombinant and wild type AOaV-1 isolates replicated at high titer in eggs (≥28 HAU/mL). The evaluation of the viral replication in the presence of exogenous proteases in Vero cells indicated that rAOaV-1 expressing codon optimized S gene replicated at the level comparable to wild type AOaV-1 (Figure 1E). The expression analysis of the S protein by Western blot indicated a potent expression of the full-length S protein in rAOaV-1-SARS-CoV-2-infected cells whereas expression of the HN proteins was detected in both recombinant and wt AOaV-1-infected cells (Figure 1F). These results confirm that the expression of the transgene (S) did not interfere with the growth characteristics of the AOaV-1 and could be a replication competitive vaccine candidate.

### 3.3. Exogenous Trypsin Independent Growth Characteristics of Vaccine Construct

**Figure 2:**
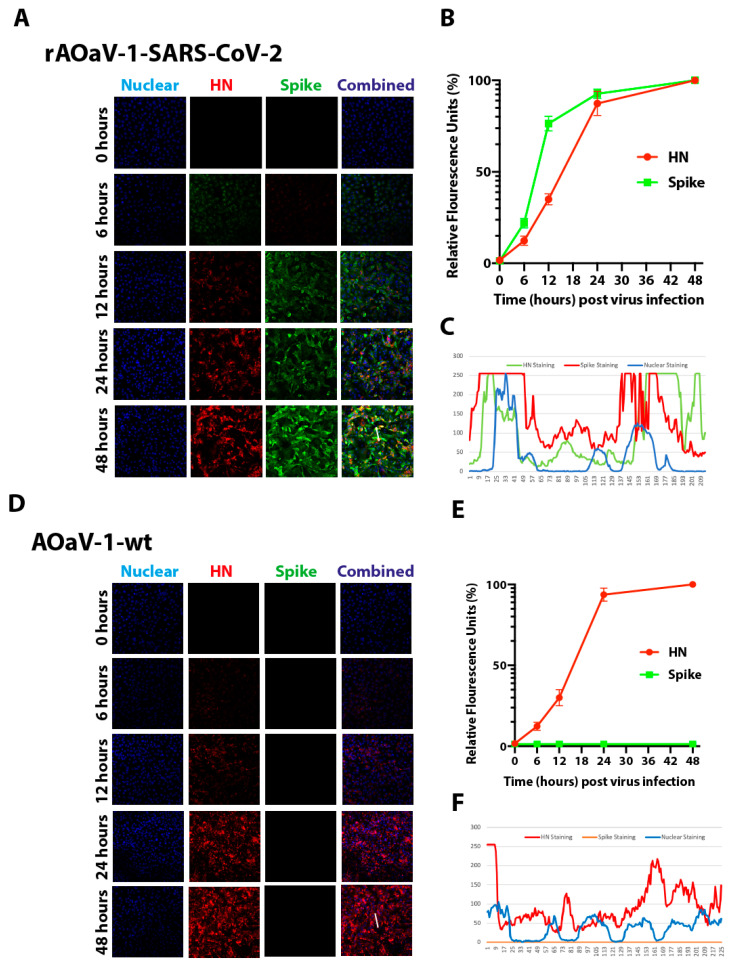
Replication of rAOaV-1-SARS-CoV-2 and AOaV-1-wt in mammalian cells. (**A**) Vero cells were infected with MOI of 1.0 with rAOaV-1-SARS-CoV-2. These cells were fixed with paraformaldehyde at 0, 6, 12, 24 and 48 h post-infection and stained for the HN protein of rAOaV-1 and S protein in the rAOaV-1-SARS-CoV-2. (**B**) Cumulative quantification of the green (S) and red (HN) fluorescence intensities before confocal microscopic imaging. (**C**) The co-expression profile for the HN and S proteins. (**D**) Vero cells were infected with MOI of 1.0 with rAOaV-1-wt. These cells were fixed with paraformaldehyde at 0, 6, 12, 24 and 48 h post-infection and stained for the HN protein of rAOaV-1. (**E**) Cumulative quantification of the green (S) and red (HN) fluorescence intensities before confocal microscopic imaging. (**F**) The co-expression profile for the HN and S proteins are graphically presented.

To initiate virus replication, the F protein of the AOaV-1 has to be cleaved by cellular proteases2. In order to investigate the pre-requisite of exogenous trypsin-like extracellular proteases for the infectivity of AOaV-1, eggs-propagated rAOaV-1-SARS-CoV-2 and AOaV-1-wt were used to infect Vero cells with a multiplicity of infection (MOI) of 1.0 without exogenous trypsin treatment. As expected, the replication of rAOaV-1-SARS-CoV-2 was apparent 6 h post-infection and the individually infected cells spread the infection to neighboring cells within the next 12 h. At 2 days post-infection, as high as 90% of the cells were infected with the rAOaV-1-SARS-CoV-2 and the majority of cells co-expressed HN protein of the AOaV-1 and S protein of the SARS-CoV-2 (Figure 2A and Figure S2). Cumulative fluorescence dynamics, based on either HN or S protein expressions, over the course of the infection, confirmed a gradual spread of the infection (Figure 2B). Co-expression fluorescence profile highlighted the co-localization of both surface proteins (Figure 2C). Similar to recombinant AOaV-1, the AOaV-1-wt replicated to a similar extent over the course of two days post-infection without the need of exogenous extracellular proteases and saturated levels of expression of the HN protein was observed at 48 h post-infection (Figure 2D and Figure S2). The cumulative fluorescence dynamics (Figure 2E) and fluorescence profile (Figure 2F) confirmed the active and progressive expression and HN-specific staining, respectively. Taken together, these results confirm the active replication, and expression of AOaV-1 and foreign genes in Vero cells independently of exogenous trypsin.

### 3.4. The rAOaV-1-SARS-CoV-2 Is Sensitive to AOaV-1 and SARS-CoV-2 Antisera

**Figure 3:**
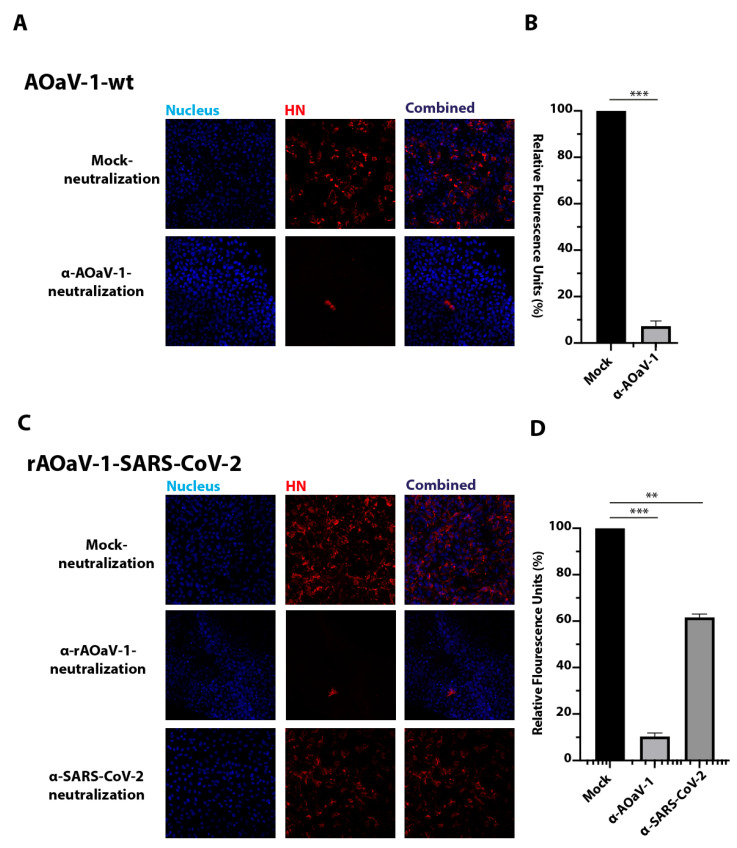
Sensitivity of recombinant and wild type (wt) viruses against antisera. (**A**) AOaV-1-wt virus was incubated with an antiserum against AOaV-1 for 2 h or was incubated with plain antiserum. The Vero cells were then infected with these viruses and incubated for 24 h before staining against the HN protein of AOaV-1. (**B**) The mock-neutralization was set to 100%, and the quantitative analysis of the virus replication was plotted for the AOaV-1 neutralized with antiserum. (**C**) rAOaV-1-SARS-CoV-2 virus was incubated with an anit-AOaV-1 antiserum or SARS-CoV-2 antiserum for 2 h before infection of Vero cells for another 24 h. These cells were then stained for the expression of the HN glycoprotein of rAOaV-1-SARS-CoV-2. (**D**) Quantitative measurement of the staining intensities plotted against the mock-treated neutralization control. *** indicates statistically significant difference with *p* < 0.005, and ** indicates level of significance at *p* < 0.05.

The F and HN surface glycoproteins are critical for receptor binding as well as membrane fusion which are indispensable for virus entry and subsequent initiation of the virus replication [37,38]. In contrast, the coronaviruses have only a single enveloped glycoprotein, which performs dual functions of receptor binding and membrane fusion [39]. In order to understand the influence of the S protein of the SARS-CoV-2 on the infectivity of the recombinant virus, the sensitivities of AOaV-1 and SARS-CoV-2 antisera were assessed and compared for neutralization. As expected, the AOaV-1-wt was resistant to mock antiserum neutralization; however, it was almost fully neutralized by the serum against the AOaV-1 (Figure 3A). Quantitatively, the anti-AOaV-1 antiserum reduced the infection of AOaV-1-wt in Vero cells by ~92% compared to the mock-neutralization (Figure 3B). In contrast, neutralization analysis of the rAOaV-1-SARS-CoV-2 showed a significant blockage of the virus entry by pre-incubation and subsequent infection of rAOaV-1-SARS-CoV-2 with either anti-AOaV-1 or anti-SARS-CoV-2 anti-sera (Figure 3C). There was no neutralization observed upon the virus’s treatment with the naïve mouse control serum. However, compared to mock-neutralization, a total of ~90% inhibition of the rAOaV-1-SARS-CoV-2 was observed with anti-AOaV-1 antiserum and ~40% inhibition was noticed with anti-SARS-CoV-2 anti-serum (Figure 3D). These observations confirm that the incorporation of S protein of SARS-CoV-2 into the recombinant AOaV-1 particles attribute to the sensitivity of the AOaV-1 to anti-SARS-CoV-2 antiserum and anti-AOaV-1 antiserum.

### 3.5. In Vitro Growth Characterization of the Vaccine Construct

**Figure 4:**
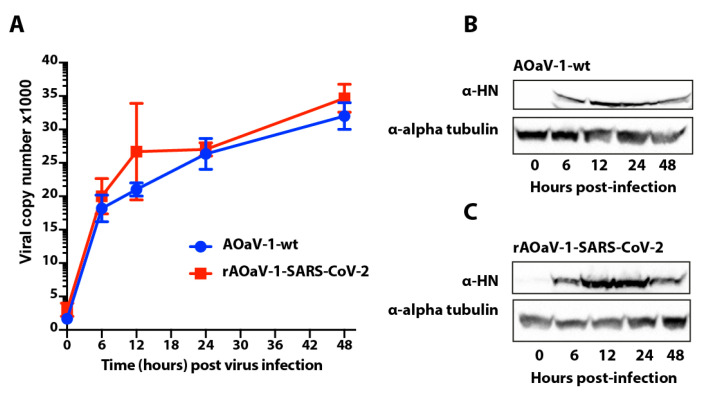
Growth kinetics of the rAOaV-1 SARS-CoV-2 vaccine and AOaV-1 wt constructs. (**A**) Vero cells were infected with an MOI 1.0 of rAOaV-1-SARS-CoV-2 or AOaV-1-wt for 2 h (0 h post infection) and were then replaced with media to be incubated and extraction of RNA at 6, 12, 24 and 48 h post infection. The viral copy numbers were calculated from AOaV-1 standard run in parallel. (**B**) Vero cells were infected with AOaV-1 and total cell lysate was prepared at indicated time points post-infection. These lysates were run for Western blotting using HN antibodies to demonstrate virus replication and alpha tubulin as loading control. (**C**) Similar to section B, cells were infected with rAOaV-1-SARS-CoV-2 and the expression for the HN and alpha tubulin was measured at indicated time points.

In order to understand the replication competence of rAOaV-1-SARS-CoV-2 and AOaV-1-wt, multistep growth kinetics was evaluated in Vero cells. The time-course quantitative measurement of the genomic copies confirmed that the expression of the S gene did not interfere with the viral replication and the rAOaV-1-SARS-CoV-2 replicated competitively and comparability with the AOaV-1-wt (Figure 4A). The cell lysate from the same experimental setting was used to assess the expression of the AOaV-1 structural proteins. The expression analysis using Western blotting demonstrated that both AOaV-1-wt (Figure 4B) and rAOaV-1-SARS-CoV-2 (Figure 4C) progressively replicated in Vero cells and expressed the HN protein as early as 6 h post-infection and as late as 2 days after initiation of the infection. These results demonstrated that the expression of the foreign genes by the AOaV-1 was unable to further attenuate the replication of the virus and could yield into sufficient viral quantity in cell culture system.

### 3.6. Stability of Recombinant AOaV-1 SARS-CoV-2 Vaccine Candidate

**Figure 5:**
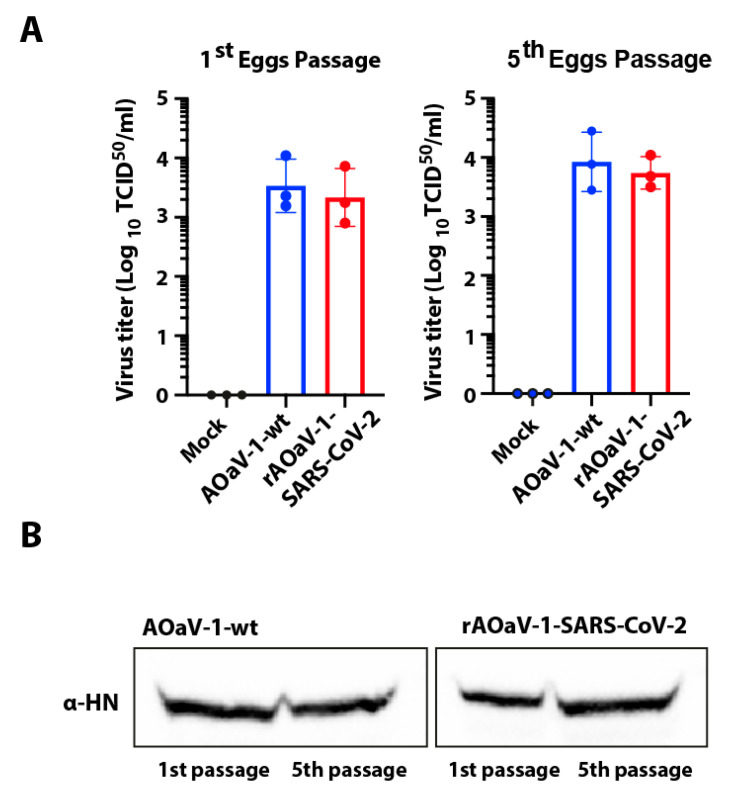
Stability replication of AOaV-1 SARS-CoV-2 vaccine candidate in embryonated chicken eggs. (**A**) The rAOaV-1-SARS-CoV-2 as well as rAOaV-1-wt were propagated in eggs and were quantified in Vero cells. The titer quantification, as shown in the bar chart, represents comparable replication. Both rAOaV-1-SARS-CoV-2 and rAOaV-1-wt were consecutively propagated in chicken embryonated eggs for 5 passages and the virus titer was quantified in Vero cells. (**B**) The first and fifth passages for both rAOaV-1-SARS-CoV-2 and rAOaV-1-wt were used to infect Vero cells for 24 h before cell lysis and expression analysis for the HN structural protein of the rAOaV-1.

In order to assess the stability of the foreign gene in the recombinant AOaV-1, the recovered and S-gene expression-confirmed viruses were passaged in 8-day-old embryonated chicken eggs for five consecutive passages. Both rAOaV-1-SARS-CoV-2 and AOaV-1-wt replicated substantially in eggs (≥28 HAU/mL). The comparative replication competence was assessed between first and fifth egg-passaged viruses in Vero cells. The rAOaV-1-SARS-CoV-2 expressing the S protein of the SARS-CoV-2 grew efficiently and at the level of the AOaV-1-wt in the first passage as well as after fifth passage in the embryonated eggs (Figure 5A). Correspondingly, the expression of the structural protein of the AOaV-1 further confirmed the stable propagation of the wt and recombinant viruses at least for several passages (Figure 5B). The sequence integrity of the inserted S gene as well as the P and M junction was assessed without any mutations. Additionally, sequencing of the S gene from the first and fifth passages confirmed no unwanted mutations in purified viruses. These results demonstrate that the rAOaV-1-SARS-CoV-2 expresses S gene stably and this expression did not significantly impact the in vitro growth characteristics of the recombinants.

## 4. Discussion

The precipitously increasing deaths, negative impact on lives and livelihood, and trembling economies warrant the urgent development of SARS-CoV-2 vaccine which would be safe, efficacious and scalable. Amongst experimental vaccines being presented for SARS-CoV-2, the vectored-based vaccines hold potential for an effective vaccine against CoVID-19 [4]. However, each viral vector inherits multiple advantages and disadvantages and careful consideration of gene delivery system may pave the way for an effective vaccine.

We propose a pre-tested vaccine vector based on the recombinant apathogenic strain of AOaV-1 (i.e., NDV). We engineered AOaV-1 to express the full-length S glycoprotein of SARS-CoV-2, which is a vital viral neutralization and major protective antigen of the virus [39]. Using exhaustive in vitro characterization of recombinant vaccine candidate, rAOaV-1-SARS-CoV-2 demonstrated efficient co-expression of surface proteins of AOaV-1 and SARS-CoV-2 in the mammalian cell line. This host-range restricting replication in mammals is one of the most attractive properties of AOaV-1 [40]. While the mechanism of host range-restriction needs investigation, it is known that AOaV-1 induces a strong interferon response in mammalian cells which in turn limits its replication [41,42]. Additionally, sialic acid receptors for the viral attachment might show fundamental differences between avian and mammalian hosts and thus defines the host restriction. It has been observed that bovine parainfluenza virus type 3, another paramyxovirus in the same family, determines the host range restriction through multiple viral proteins [43]. Notwithstanding, AOaV-1 are clearly highly restricted to primates and this selective replication is irrespective of any of the known pathotypes such as mesogenic or lentogenic strains of AOaV-1 [23]. 

In addition to the above mentioned factors, the AOaV-1 carries a range of advantages over other multiple vectors. AOaV-1 are antigenically distinct from viruses that are known to naturally infect humans and AOaV-1 does not show cross-reactivities with antibodies raised against other paramyxoviruses. Therefore, AOaV-1 induces strong immune responses in the human population. Pertinent to this feature and interferon sensitivity, AOaV-1 are considered a valuable oncolytic agent and thus propose safety in humans upon parenteral administration [44]; www.cancer.gov/cancertopics/pdq/cam/NDV/healthprofessional. Potential seroconversion and a transient conjunctivitis may occur in poultry health workers, which subsides within four days without any additional or systemic symptoms [45]. Altogether, AOaV-1 presents a pre-tested and proven safety profile in humans and may highlight speeding rolling out of the recombinant vaccines in the general human population.

Majority of vaccines (i.e., recombinant protein-based) require tedious manufacturing procedures and are therefore resource demanding. On the other hand, preparation of inactivated whole virus SARS-CoV-2 vaccines require biosafety level (BSL)-3 facilities and due to limited BSL-3 facilities preparation of such vaccines are inconvenient [5,46]. The AOaV-1-based vectored vaccines propose solutions to the abovementioned limitation of subunit vaccines with high titer replication in chicken embryonated eggs which are low-resource demanding and may be scaled up in developing countries. Additionally, using the existing influenza virus vaccine production pipelines, AOaV-1-based vaccines can be manufactured under BSL-2 conditions. Importantly, our results also demonstrated that a AOaV-1-based CoVID-19 vaccine candidate can also be propagated in Vero cells which is a US FDA-approved cell line for human vaccine production. 

Similar to other paramyxoviruses, AOaV-1 enters host cells by direct fusion at the plasma membrane through a pH-independent mechanism [40,41]. The AOaV-1 can also enter host cells by an endocytic pathway. The entry of the AOaV-1 into the cell is then mediated by the surface fusion (F) glycoprotein, which is also a major determinant of AOaV-1 virulence in birds [42]. The viral infectivity requires cleavage of the F protein through the intracellular ubiquitous proteases including furin, allowing disseminated replication in multiple organs and tissues. By a mechanism similar to AOaV-1, rAOaV-1-SARS-CoV-2 enter the cells through proteolytic cleavage of the S protein [39]. The trypsin-independent infectivity of rAOaV-1-SARS-CoV-2, as was observed in our study, facilitated the vector propagation in the cell-culture model as well as embryonated chicken eggs.

A wealth of data has been generated on the safety, efficacy and immune-regulatory properties of AOaVs as oncolytic agent in humans. Specifically, it has been demonstrated that AOaVs show profound stimulation of innate immunity and production of multiple cytokines, such as IFN-α, IFN-β, TNFα and IL-1, which in turn activate NK cells, macrophages, and sensitized T cells [25,46,47,48]. Additionally, AOaVs are known to upregulate major histocompatibility complex (MHC) class I molecules and enhance T-cell co-stimulatory activities which results in enhanced effector cells [49,50]. A genetically modified AOaVs expressing human IL-2 resulted in the activation of cytotoxic T lymphocyte (CTL) and memory T cells [51]. Collectively, owing to pleiotropic immunostimulatory effects, T helper (T_H_) responses, and CTL, NK cells, and macrophages activation, AOaVs show promising safety and immunogenicity profiles as vaccine vector.

It has previously been shown that insertion of the transgene may make the vector sensitive to neutralization by antibodies, which are specific to the inserted protein and may facilitate the seepage of neutralization by vector-specific neutralizing antibodies [52]. The rAOaV-1-SARS-CoV-2 expresses both the native structural HN protein as well as S glycoprotein and therefore the entry into the cell may be attributed to the AOaV-1-like or SARS-CoV-2-like property. In conjunction with this hypothesis, the anti-AOaV-1 antiserum, as well as the anti-SARS-CoV-2, could substantially block the entry of the rAOaV-1-SARS-CoV-2. Additionally, the recombinant AOaV-1-SARS-CoV-2 offers an exciting system to underpin functional interactions between native and foreign envelope glycoproteins in one viral particle. 

Recently, insertion of the foreign genes into the backbone of the AOaV-1 has been practiced for multiple viruses including influenza, SARS, human immunodeficiency virus [13], human parainfluenza, rabies [15], Nipah disease [16], Rift Valley fever [17], Ebola and highly pathogenic H5N1 [18,19]. In several studies, it has been demonstrated that insertion of the transgene into the genomes of AOaV-1 resulted in reduced pathogenicity in poultry birds. This safety was maintained even after the expression of the HA gene from a highly pathogenic avian influenza virus [18,19]. Sustained, stable and progressive replication of both wt and recombinant viruses demonstrated effective independent spread within cells.

## 5. Conclusions

Taken together, our results demonstrated that the recombinant vector expressing S protein propagated stably in chicken embryonated eggs for several consecutive passages, and this expression did not significantly impact the in vitro growth characteristics of the recombinants. The presented respiratory vaccine candidate has the potential for further development as vaccine vector to be available for expedited vaccine development for pre-clinical and clinical studies.

## Supplementary Materials

The following are available online at https://www.mdpi.com/2076-393X/8/3/472/s1: Figure S1: A real-time PCR-based detection of rescued rAOaV-1-wt (A) and rAOaV-1-SARS-CoV-2 (B) in chicken embryonated eggs. (C) Quantitative presentation of rAOaV-1-wt or rAOaV-1-SARS-CoV-2 detection. (D) Hemagglutination assay used to screen the chicken embryonated eggs for the presence (+)/absence (–) of rAOaV-1-wt or rAOaV-1-SARS-CoV-2, Figure S2: Replication of wt and recombinant viruses in Vero cells. (A) Comprehensive and expanded confocal microscopic images representing Figure 2A–C. (B) Comprehensive and expanded confocal microscopic images representing Figure 2D–F.
