# Supplementary material for: A Scalable Topical Vectored Vaccine Candidate against SARS-CoV-2
Source: Vaccines (Basel). 2020 Aug 24;8(3):472. doi: 10.3390/vaccines8030472 (PMC7565466; doi:10.3390/vaccines8030472)
Supplement: Supplementary file 1 [file vaccines-08-00472-s001.pdf]

A Scalable Topical Vectored Vaccine Candidate Against SARS-CoV-2

Mohammed A Rohaim and Muhammad Munir*

Division of Biomedical and Life Sciences, Faculty of Health and Medicine, Lancaster University, Lancaster LA1 4YG, UK

*Correspondence: Email: muhammad.munir@lancaster.ac.uk; Tel: +44 (0)1524 595083

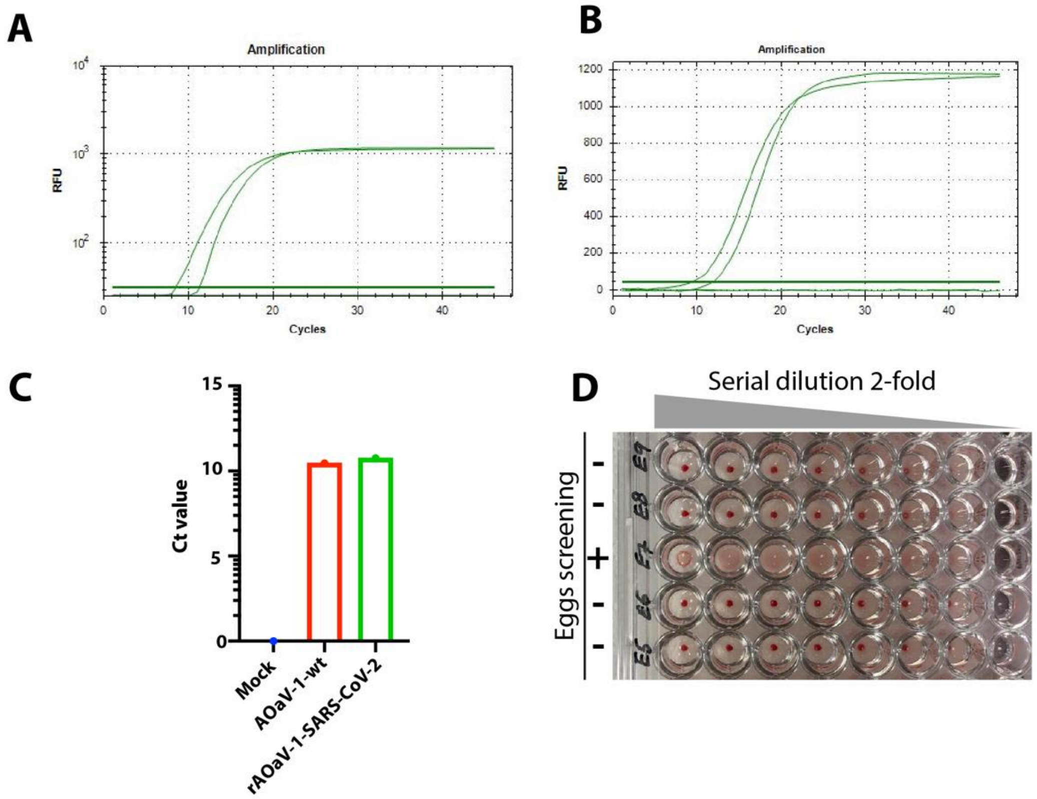

Figure S1. A real-time PCR-based detection of rescued AOaV-1-wt (**A**) and rAOaV-1-SARS-CoV-2 (**B**) in chicken embryonated eggs. (**C**) Quantitative presentation of AOaV-1-wt or rAOaV-1-SARS-CoV-2 detection. (**D**) Hemagglutination assay used to screen the chicken embryonated eggs for the presence (+)/absence (-) of AOaV-1-wt or rAOaV-1-SARS-CoV-2.

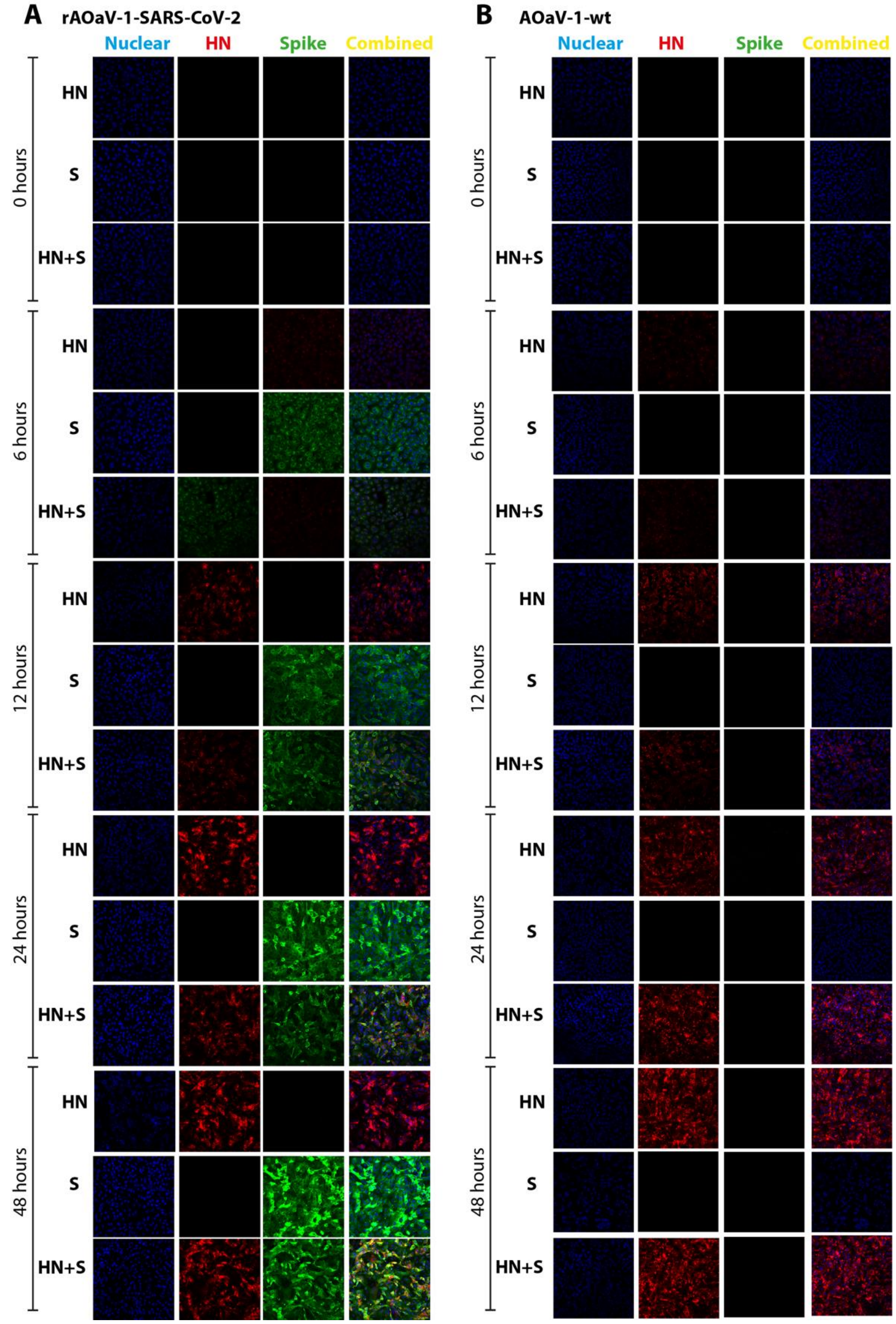

Figure S2. Replication of wt and recombinant viruses in Vero cells. (**A**) Expanded confocal microscopic images representing Figure 2 A-C in the main text. (**B**) Comprehensive and expanded confocal microscopic images representing Figure 2 D-F in the main text.
